# Perennial vegetables: A neglected resource for biodiversity, carbon sequestration, and nutrition

**DOI:** 10.1371/journal.pone.0234611

**Published:** 2020-07-10

**Authors:** Eric Toensmeier, Rafter Ferguson, Mamta Mehra

**Affiliations:** 1 Perennial Agriculture Institute, Holyoke, Massachusetts, United States of America; 2 Union of Concerned Scientists, Washington, D.C., United States of America; 3 Perennial Agriculture Institute, Holyoke, Massachusetts, United States of America; Banaras Hindu University, INDIA

## Abstract

Perennial vegetables are a neglected and underutilized class of crops with potential to address 21^st^ century challenges. They represent 33–56% of cultivated vegetable species, and occupy 6% of world vegetable cropland. Despite their distinct relevance to climate change mitigation and nutritional security, perennial vegetables receive little attention in the scientific literature. Compared to widely grown and marketed vegetable crops, many perennial vegetables show higher levels of key nutrients needed to address deficiencies. Trees with edible leaves are the group of vegetables with the highest levels of these key nutrients. Individual “multi-nutrient” species are identified with very high levels of multiple nutrients for addressing deficiencies. This paper reports on the synthesis and meta-analysis of a heretofore fragmented global literature on 613 cultivated perennial vegetables, representing 107 botanical families from every inhabited continent, in order to characterize the extent and potential of this class of crops. Carbon sequestration potential from new adoption of perennial vegetables is estimated at 22.7–280.6 MMT CO2-eq/yr on 4.6–26.4 Mha by 2050.

## Introduction

The perennialization of crop production has been proposed as a multifunctional approach to address environmental and other challenges in agriculture, due to the many benefits of perennial crops [1], but the perennialization of vegetable production has largely been ignored. Perennial vegetables (PVs) are a neglected and underutilized class of crops with potential to address crises of crop biodiversity, climate change, and nutrient deficiencies. While some individual species have been studied closely, as a class PVs have received little attention in peer-reviewed literature (for exceptions see [1–3]), though a body of grey literature has developed on the subject in recent decades [4, 5].

PVs are perennial plants cultivated for their edible vegetative growth (e.g., leaves) and/or reproductive structures (e.g., flowerbuds). They include some savory tree fruits that are used in cooked dishes, but not sweet or tart dessert fruits. Further definition is offered in the Methods below.

Many PVs are suited to conditions where production of annual vegetables is difficult. For example, PV crops include numerous halophytes, desert species, aquatic species, and shade crops. The many shade tolerant PVs are particularly suited to multistrata agroforestry systems [4].

While many PVs are regional crops, of which little is known outside of the areas in which they are cultivated, a few are widely known and globally traded commodity crops. PVs are currently cultivated on at least 3.3 Mha, 6% of the 52 Mha of world vegetable land. FAO [6] reports 1.1 Mha in table olives (*Olea europaea*, 10.6 Mha in global production, of which 10% is used for table olives, here defined as a vegetable use, [7]), 1.5 Mha in asparagus (*Asparagus officinalis*), 0.6 Mha in avocado (*Persea americana*), and 0.1 Mha in globe artichoke (*Cynara scolymus*). Additionally, India has 38,000 ha in the tree vegetable moringa (*Moringa oleifera*, [8]). Note that these are only five of the over 600 cultivated species inventoried in this paper, though the area in cultivation of most PVs is surely very small.

Greater adoption of a wider array of perennial vegetables could help to address some of the central, interlocking issues of the 21^st^ century: climate change, biodiversity, and nutrition. The great diversity of PVs is a powerful tool to address the loss of crop biodiversity. As perennials, PVs sequester carbon, particularly the woody species. Many PVs are high in the key nutrients needed to remedy nutrient deficiencies that impact billions of people.

### Crop biodiversity

Crop biodiversity is essential to agroecological food production and multifunctional agriculture. However, globally, crop biodiversity is declining, in part driven by intensive, industrialized agriculture [9] Eighty percent of global crop production comes from 17 botanical families, just 4% of the total number of plant families [10].

Many benefits can come from increasing crop biodiversity. Increased cultivation of neglected and underutilized perennial crops can increase resilience to climate change impacts. This kind of crop diversification is an important risk-management strategy, especially in the context of the narrowing of crop diversity on which food security depends. Many perennial crops produce in seasons when other crops are not available [11]. Crop diversification at the family level can also reduce pest pressure, as many pests and diseases are family-specific [12].

### Climate change mitigation

Agricultural production is the source of 12.5% of anthropogenic emissions, yet the agriculture sector also has mitigation potential, including via carbon sequestration [13]. The perennialization of agriculture is one way to sequester excess atmospheric carbon in soils and living biomass. This includes transition to reduced tillage perennial crops and use of agroforestry systems which incorporate perennial plants [14]. PVs are well-suited to no-till and agroforestry production systems as they do not require tillage after establishment, and many are shade-tolerant, making them ideal for the understory of agroforestry systems. For example, in western China, the PV species daylily (*Hemerocallis fulva*) is cultivated for its edible flowers in the understory of rows of jujube (*Zizyphus jujuba*), alternating with rows of annual crops [15].

### Nutrition

Recent research has shown that for all of the world’s people to consume a healthy portion of vegetables in their diet, global vegetable production would need to be tripled [16]. While underconsumption of vegetables is a global problem, economic and development context produce distinct syndromes of nutrient deficiency: what is sometimes called “traditional” malnutrition (largely in the Global South), and industrial diet deficiencies (largely in the Global North) [17].

An estimated two billion people are affected by traditional malnutrition, a set of deficiencies of particular vitamins and minerals. The most significant of these deficiencies are iron, zinc, vitamin A, iodine, and folate. Collectively deficiencies in these nutrients account for 7% of global disease burden [18]. While iodine is not found at useful levels in terrestrial plants, iron, vitamin A, zinc and folate are found at high levels in some vegetables including PVs.

Meanwhile, the low vegetable intake characteristic of the Western industrial diet produces its own separate set of nutritional deficiencies, impacting hundreds of millions of people and increasing the prevalence of heart disease, osteoporosis, high blood pressure, diabetes, and obesity. The key nutrients in this case include fiber, calcium, magnesium, and antioxidants including vitamins A, C, and E [17]. Magnesium deficiency affects 60% of people in the United States, and is implicated in diabetes, high blood pressure, and heart disease [19]. Calcium is critical to reducing risks from osteoporosis [20]. Dietary fiber is correlated with reduction in coronary heart disease and obesity. In the United States average fiber intake is less than 50% of the recommended levels [21]. Antioxidants help to reduce the risk of cardiovascular disease, a widespread health issue in wealthy countries [22]. Many PVs are quite high in fiber, calcium, magnesium, and antioxidants and thus have potential to address these industrial diet deficiencies.

### A role for perennial vegetables

Given the relevance of PVs to the interlocking crises of crop biodiversity loss, climate change, and nutrient deficiencies, it is imperative to explore the potential of this underutilized and neglected class of vegetables. In order to address the scope of this resource and its potential contribution to addressing the agricultural biodiversity crisis, we must ask how many PV species are cultivated, and what agroecological niches they occupy in regards to form, parts used, and climate suitability. To investigate their climate mitigation potential, this study investigates the per-hectare carbon sequestration potential of PVs, as well as asking how widely they might be adopted in the future. To determine the nutritional impact of PVs, this study asks how PVs compare to globally-traded vegetables, and seeks standout species both “suberabundant” in individual key nutrients as well as “multi-nutrient” crops with high levels of two or more nutrients essential to addressing deficiencies. The study also investigates the relative nutrition of different forms (e.g. woody plants) and parts used (e.g. flowerbuds).

## Materials and methods

### Perennial vegetables defined

As PVs are a class of crops that has received little notice, we begin by offering a functional definition. The criteria for this definition include botanical form, harvest considerations, what parts used, and how those parts are used in the world’s cuisines.

For purposes of this study, “perennial” indicates crops which live for 3 or more years. We divide the PVs discussed in this study into three categories by form: woody plants, vines, and herbs. Woody plants include trees, shrubs, bamboos, palms, cacti, mangroves, and woody succulents. Perennial vines include both lianas and herbaceous vines. Perennial herbs include forbs, ferns, grasses and grasslike plants, and both floating and emergent aquatics. For the purposes of this discussion we include perennial crops that are sometimes cultivated as annuals. For example, the African eggplant (*Solanum aethiopicum*) is most commonly grown as an annual but can also be managed as a perennial. We include some species which include both perennial and annual (or biennial) crop varieties, such as kale (*Brassica oleracea*).

In order to be considered perennial, vegetables must provide more than one year of harvest. We exclude crops that live for several years but are killed by harvest, such as with some heart-of-palm vegetables like coconut [23].

Some PVs have edible vegetative parts. This includes leaves, shoots, and other vegetative structures like petioles and cactus cladodes. Culinary herbs are excluded based on the assumption that they are consumed in smaller quantities than vegetables due to strong flavors. Some species are used as culinary herbs in one region, and vegetables in another. In such cases, the crop is included in this study’s analysis.

Other PVs have edible reproductive structures, including flowerbuds, flowers, unripe fruit, ripe fruit, and unripe seeds. Classification of fruits as vegetables is challenging. Many annual fruits are commonly used as vegetables, such as tomato (*Solanum lycopersicum*), string beans (*Phaseolus vulgaris*), and winter squash (*Cucurbita* spp.). The distinction is not botanical but rather based on how the fruit is used and how it tastes. Use also varies to some degree between cultures. This study follows [24] in distinguishing between dessert fruits which are sweet or tart, and vegetable fruits which are eaten in salads, cooked dishes, and appetizers. Dessert fruits are excluded from the study. PV fruits are used in salads, cooked in soups and stews, or otherwise a central part of a meal. For example, the fruit of chayote (*Sechium edule*) is widely consumed as a vegetable in Mesoamerican cuisines and throughout the tropics. In some cases a fruit is a vegetable when unripe, and a dessert fruit when ripe, as in the papaya (*Carica papaya*).

Roots crops like sweet potato (*Ipomoea batatas*) and starchy fruits like breadfruit (*Artocarpus altilis*) are excluded on the grounds that nutritionally they are more properly seen as staple crops primarily producing carbohydrates, with lower vitamin and mineral content than other vegetables. Root crops are also incompatible with no-till perennial production systems as they require excavation for harvest [13].

### Biodiversity

This study aims to quantify for the first time how many PV species are in cultivation, and identify their agroecological niches as to climate, shade tolerance, form and parts used. In the absence of a well-integrated literature, several approaches were used to identify and characterize the global extent of cultivated perennial vegetable crop species.

The first approach analyzed species from [25], which provides a global list of cultivated vegetables. Though incomplete, it is international and can be used as a proxy for the world’s vegetable crop biodiversity. After the elimination of root crops (as above), the remaining species were categorized into perennial, perennial grown as annual, and annual crops.

Second, we reviewed 6,000 species profiled in [26], a six-volume compendium of cultivated crops (excluding ornamentals), identifying those cultivated as vegetables. All species that are cultivated for the purpose of vegetable production, whether or not it is their primary use, were considered for this study. Note that cultivation does not necessarily indicate domestication.

The third approach was a search for cultivated PVs in global listings of crops and vegetables, as well as regional and specialty resources. The resources used in developing this listing were [4, 5, 13, 26–53]. These crops were classified by part used, form, cultivation status, and adaptability to climate, moisture, and shade. This information is important in assessing the PV species available to fit various agroecological niches.

All cultivated PVs identified by the study were characterized by climate suitability and shade tolerance. Thermal climate analysis divided the crops into tropical lowlands (USDA hardiness zones 10–11, elevation under 1500m), tropical highlands (USDA hardiness zones 10–11, elevation over 1500m), subtropical (USDA zone 9), warm temperate (USDA zones 7–8), cold temperate (USDA zones 4–6), boreal (USDA zones 2–3), and arctic (USDA zone 1). Rainfall categories are humid (1000+mm rainfall/yr), semi-arid (250-1000mm rainfall/yr), arid (0-250mm rainfaal/yr), and aquatic. Shade tolerance was also assessed.

### Carbon sequestration

In order to assess the potential carbon sequestration potential of PVs, we must first estimate per-hectare sequestration rates, adoption rates, and total potential adoption. Existing data are sparse for all aspects of this question. Despite this, an approach based on available data and a range of plausible scenarios is useful for exploring the and defining the boundaries of the solution space for these critical questions. In order to model potential impact, we therefore developed twelve scenarios built around estimates for carbon sequestration rate, and a range of plausible values for rate of adoption, potential (maximum) scale of adoption, and the ratio of adoption between woody and herbaceous PVs.

We consulted the research literature for the available data on per-hectare carbon sequestration impacts of individual PVs, as well as sequestration rates for relevant farming systems (Table 1), categorized by a three-level classification scheme: a broad top-level dichotomy of (1) woody perennials and (2) perennial vines and herbs, and then production system and sample species or system. The average rate for woody PVs is 3.7 tC/ha/yr, while that of perennial vines and herbs is 0.43 tC/ha/yr/.

**Table 1:** Carbon sequestration rates of PVs.

PV Category	PV production system type	Sample species or system	Sequestration rate MgC/ha/yr	Source
Woody perennial crops	Orchard and plantation; full-sized woody plants for flowers, fruits, and unripe seeds	Convert cropland to orchard	3.5	[54]
		*Olea europaea*	2.6	[55]
		*Bactris gasipaes*	5.1	[56]
		*Dacryodes edulis*	7.8	[57]
		Tree crops–temperate	2.1	[58]
		Tree crops–tropical	1.8–10.0	[58]
	Bamboo for shoot production	Bamboo plantations	6.0–13.0	[59]
	Coppiced woody plants for edible leaves	Fodder tree blocks	0.1–0.5	[54]
		Short rotation coppice	1.18	[60]
	***Average sequestration rate***		**3.71**	
Perennial vines and herbaceous crops	Perennial vines	*Vitis vinifera*	0.3–0.8	[61]
	Robust perennial herbs (over 2m height)	Giant biomass grasses	1	[62]
		Perennial grains	0.3–0.5	[63]
	Ordinary perennial herbs (under 2m height)	Residential landscape with herbaceous perennials	0.0–0.1	[64]
	***Average sequestration rate***		**0.43**	

Carbon sequestration rates of PVs, where known, are shown here. Rates for farming systems that match PV production systems are shown as well.

The ratio of Mha in woody to non-woody PVs is important to scenario development, as woody PVs have much higher sequestration rates. Currently there at 1.7 Mha of woody Pvs and 1.6 Mha of herbaceous perennial PVs [16]. The scenarios assume that 25%, 50%, and 75% of new adoption is woody.

The potential scale of adoption of PVs is also necessary to calculate climate impact. One set of scenarios assumes that total global vegetable production area remains constant at 58.2 Mha. A second set of scenarios assume that world vegetable production area triples to 174.5 Mha, in line with estimates of the growth needed to provide all of humanity with sufficient nutrients [16].

Adoption rates are set based on historical data from [6] for the global production area of artichoke, asparagus, avocado, and olive from 1967–2017. In the absence of similar data for the other PVs, only the sum of these four PVs is considered in projecting the future adoption between 2020–2050. Both linear and exponential trends are modeled.

Twelve scenarios were developed, using the variables adoption rate (linear and exponential), percent of woody PVs out of total PVs adopted (25%, 50%, and 75%), and total global vegetable production area (58.2 Mha and 174.5 Mha). In scenarios 1 and 2, the current world vegetable production area is used, while in scenarios 3 and 4, world vegetable production area is tripled to meet nutritional needs. In scenarios 1 and 3, a linear adoption rate is used, while in scenarios 2 and 4 an exponential rate is used. For each scenario, woody PVs are assumed to occupy 25%, 50%, and 75% of new adoption.

### Nutrition

Determining the potential of PVs to address nutrient deficiencies is a core goal of this study. To this end, it aims to compare PVs to globally-traded vegetables in several ways. Individual species with “superabundant” levels of single key nutrients are identified, as are “multi-nutrient” species with high levels of multiple key nutrients. Subclasses of PVs, based on form and part used, are also compared to globally-traded vegetables.

Nutrients assessed were those identified as priorities for addressing deficiencies in the literature (fiber, Ca, Fe, Zn, folate, and antioxidants). In the case of antioxidants, vitamins A, C, and E were selected as data are more available for these than other antioxidants. Note that terrestrial plants do not contain significant iodine, so this nutrient was not assessed despite the importance of addressing iodine deficiency.

Following an extensive literature review, we conducted a meta-analysis on nutrient content using data from [17, 28, 32, 36, 40, 41, 49–50, 53, 65–109]. Our analysis produced mean nutrient values for 240 species of perennial vegetables, including 31 species that are frequently grown as annuals despite having a perennial lifecycle.

As the international standard for vegetable nutrient testing is a fresh weight sample of 100 grams, this was used as the basis of data collection and comparison for this study. In several cases values were converted from dry weight to fresh weight. As vegetables are usually consumed fresh (rather than dried), and this study compares vegetables to vegetables, values were not converted to dry weight, though dry weight comparison would offer some advantages.

For several reasons, the direct ability of these crops to meet daily requirements was not calculated. The first reason is that for all vegetables, limited bioavailability means that less than 100% of nutrients are actually digested. Bioavailability is variable, for example increasing if small amounts of animal protein are consumed, and this makes calculating the precise dietary impact of vegetable consumption difficult. Nevertheless there is consensus that vegetables are an essential source of nutrients [110]. The second reason is that the daily requirement for each nutrient varies widely between ages, genders, and pregnancy and lactation status [111]. It was therefore determined that the data should be compared to vegetables which are widely grown and marketed.

To this end, we selected 22 vegetable species tracked by the FAO Statistical Service [16] as a reference set and nutritional benchmark. These reference crops, both annual and perennial, include okra (*Abelmoschus esculentus*), leek (*Allium ampeloprasum*), scallion (*Allium fistulosum*), asparagus (*Asparagus officinalis*), broccoli (*Brassica oleracea* Italica group), cabbage (*Brassica oleracea* Capitata group), cauliflower (*Brassica oleracea* Botrytis group), kale and collard greens (*Brassica oleracea* Acephala group), pak choi and Chinese cabbage (*Brassica rapa*), pepper (*Capsicum annuum*), cucumber (*Cucumis sativus*), summer squash and zucchini (*Cucurbita* spp.), winter squash (*Cucurbita* spp.), globe artichoke (*Cynara scolymus*), lettuce (*Lactuca sativa*), avocado (*Persea americana*), green bean (*Phaseolus vulgaris*), pea (*Pisum sativum*), tomato (*Solanum lycopersicum*), eggplant (*Solanum melongena*), spinach (*Spinacea oleracea*), and sweet corn (*Zea mays*). While cassava leaf (*Manihot esculenta*) is also tracked by FAO, we opted to exclude it as an outlier due to its exceptionally high values. The values for summer squash/zucchini and winter squash represent the mean values for *Cucurbita moschata*, *C*. *pepo*, *C*. *maxima*, and unspecified *Cucurbita* spp, at the unripe stage for summer squash/zucchini and ripe stage for winter squash.

We constructed categorical levels for each nutrient based on nutrient levels across the set of the reference crops (Table 2). For each nutrient, we divided the range found within the reference set into “low” (the lowest third), “medium” (the middle third) and “high” (the highest third). We classified nutrient concentrations below the lowest value found in the reference vegetables as “very low,” while those above the highest value as “very high.” We classified nutrient values more than twice the highest reference crop levels “extremely high.” For Vitamin A, the lowest point in the range for the reference vegetables was 0. For this reason the “very low” category is not applied to Vitamin A.

**Table 2:** Nutrient concentration classes based on reference crop nutrient levels.

	Fiber	Ca	Fe	Mg	Zn	A	B9	C	E
	%	mg/100g	mg/100g	mg/100g	mg/100g	Mg RAE	mcg/100g	mg/100g	mg/100g
Very low	0.00–0.39	0.00–11.84	0.00–0.46	0.00–11.24	0.00–0.15	0.00	0.00–13.49	0.00–5.64	0.00–0.04
Low	0.40–1.45	11.85–86.71	0.47–1.01	11.25–35.75	0.16–0.29	0.00–0.18	13.50–73.07	5.65–42.33	0.05–0.73
Medium	1.46–2.50	86.72–161.57	1.02–1.55	35.76–60.26	0.30–0.42	0.19–0.37	73.08–132.63	42.34–79.01	0.74–1.42
High	2.51–3.85	161.58–238.70	1.56–2.11	60.27–85.50	0.43–0.56	0.38–0.55	132.64–194.00	79.02–116.80	1.43–2.54
Very high	3.59–7.15	238.71–477.40	2.12–4.21	85.51–171.00	0.57–1.12	0.56–1.11	194.01–388.00	116.81–233.59	2.55–5.08
Extremely high	7.16+	477.41+	4.22+	171.01+	1.13+	1.12+	388.01+	233.6+	5.09

“Very low” indicates nutrient values lower than the lowest value for the reference vegetables. “Low” is from the lowest tertile of the reference vegetable range, “medium” is the middle tertile, and “high” is the highest tertile. “Very high” ranges from the highest end of the range to twice the highest end, while “extremely high” is more than double the highest end of the range.

For those nutrients for which data was acquired, we compared nutrient content across categories of crop form and part used, in terms of the percentage of crops in each category with one or more superabundant nutrients. Data was not available for every nutrient for all reference crops. Similarly, data was not available for every nutrient for each PV (see S1 Fig in the supplementary materials for information on the number of data points for each nutrient, plant form, and part used). It is possible that complete data for reference crops and/or PVs would shift our results slightly.

In order to assess the potential of PV crops to ameliorate nutritional deficiencies associated with traditional malnutrition (iron, zinc, vitamin A, iodine, and folate) and industrial malnutrition (including fiber, calcium, magnesium, and antioxidants), we scored each crop separately based on the abundance of nutrients relevant to each syndrome. In both cases, “extremely high” levels were given 3 points, “very high” two points, and “high” one point. A combined score of 6 was set to qualify species as “multi-nutrient” crops.

In order to relate nutrient content with agroecological niche, we also show superabundant and multi-nutrient PVs according to the plant form and part used.

It is also a goal of this study to identify standout species with superabundant levels of individual key nutrients. To this end, the vegetables were ranked in order of concentration from highest to lowest for each nutrient. This enabled the development of “top ten” lists for each nutrient, showing the ten crops with the highest levels of the nutrient in descending order.

## Results

### Biodiversity

PVs are a large and diverse group. They represent a third to half of vegetable crop species and over 7% of all cultivated crops. Most are herbaceous perennials, but over a third are woody plants. Leaves are the most widely used part. There are species suited to virtually all climates where crops are grown.

A literature search was conducted for this study with the aim of compiling a comprehensive list of cultivated PVs. This search found 613 cultivated species of PVs. Further analysis broke down these species by cultivation status, form and part used, and climate suitability. The full list is included as supplemental document “Cultivated Perennial Vegetable Species” as an Excel spreadsheet.

We approached the goal of assessing the extent of PV cultivation in several ways. Our analysis of [25], a reference which is limited exclusively to cultivated vegetables, found 63 fully perennial crops out of a total of 180 vegetable crops, including 37 perennials sometimes grown as annuals. PVs represent 35–56% of the cultivated vegetable species profiled in this source.

This study also sought to determine the percentage of all crops which are PVs. Of the 6,000 crop species and subspecies profiled in [26], 470 are perennials cultivated as vegetables, including 45 perennials sometimes grown as annuals, suggesting that 7.7% of all cultivated crop species are PVs.

**Fig 1:**
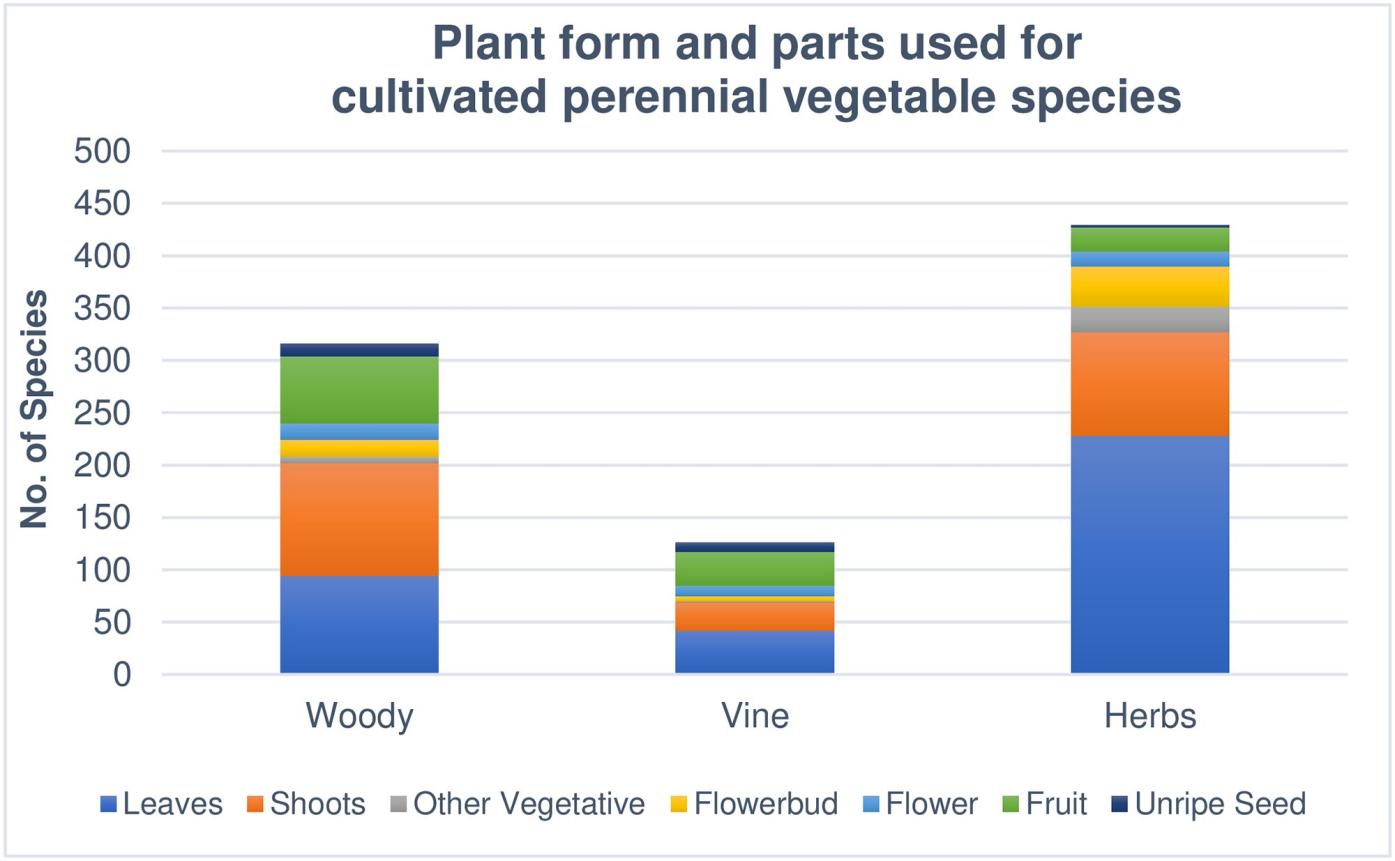
Plant form and parts used for cultivated perennial vegetable species. This figure shows the form (woody perennial, perennial vine, or perennial herb) and part consumed as vegetable (leaf, shoot, other vegetative part, flowerbud, flower, fruit, and unripe seed). Note that some species have more than one part consumed as a vegetable and thus are shown more than once.

Cultivated PV species were broken down by form and part used. Woody plants comprised 36.5%, vines 11.7%, and perennial herbs 50.9%. Of these, 114 (18.5%) are perennials often grown as annuals. Fig 1 shows the part used for woody, vine, and herbaceous perennial PVs. Note that some species have more than one part used. While 80% of global crop production comes from only 14 botanical families [10], this study found that cultivated PVs represent 107 botanical families.

**Fig 2:**
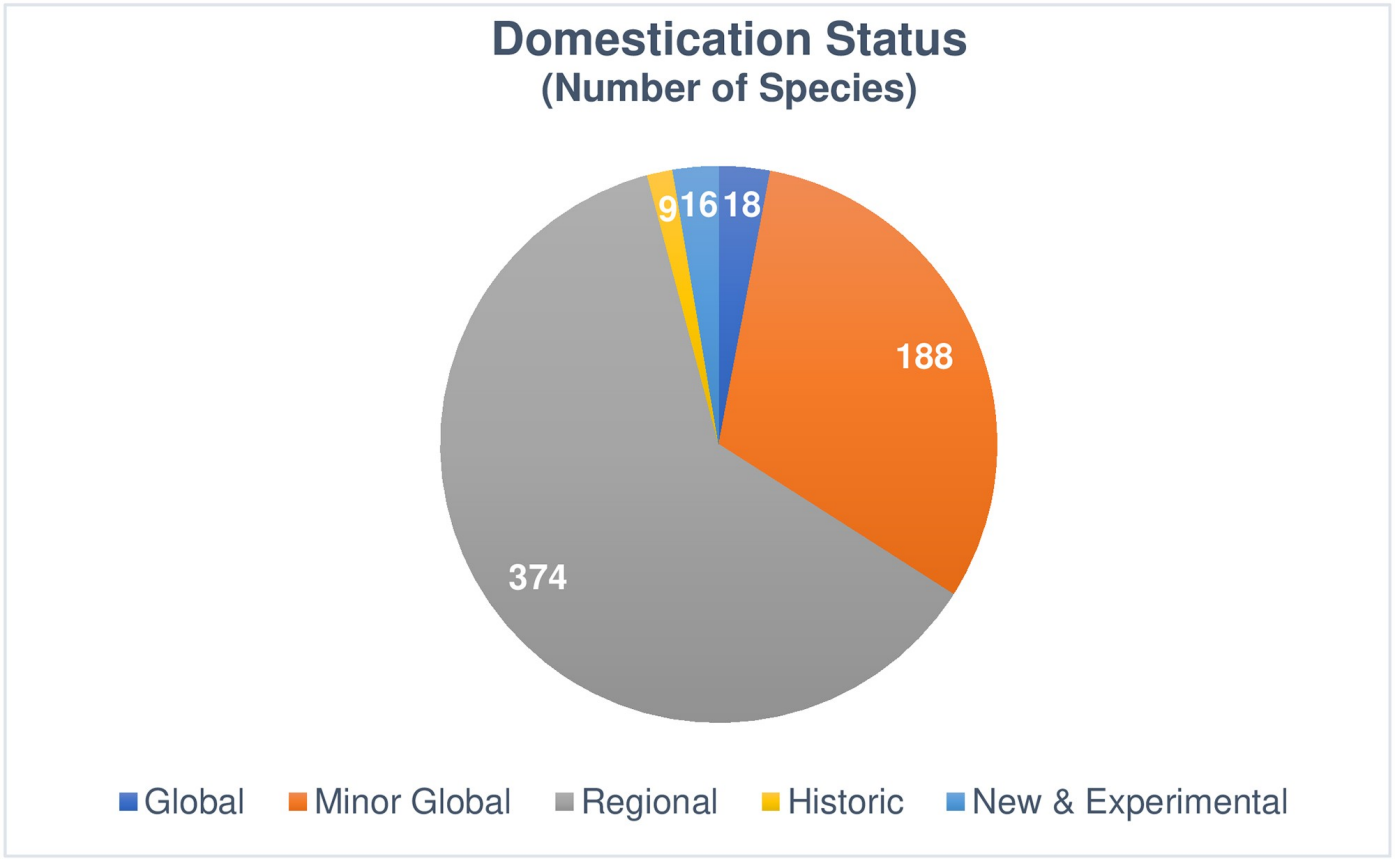
Domestication status. This figure shows the number and relative portion of cultivated PV species by domestication status. “Global” indicates produced in more than one region, with global sales over $1 billion USD. “Minor global” crops are produced in multiple regions, with value under $1 billion. “Regional” crops are cultivated only in their region of origin. “Historic” crops were cultivated historically but have been abandoned as crops. “New and experimental” crops are new to cultivation or under active breeding development.

The level of domestication of these 613 cultivated PV species was also assessed (see Fig 2). Eighteen (2.9%) are global crops, with over $1 billion USD in sales [6]. Minor global crops, which are cultivated outside of their continent of origin but with sales under $1 billion, account for 30.7% of cultivated PVs. Regional crops, which though cultivated have never been taken up outside of their region of origin, make up 61.0% of cultivated PVs. Historic crops, which are formerly cultivated but now abandoned, are 1.5%. New and experimental crops account for the remaining 2.6% of cultivated PVs.

**Fig 3:**
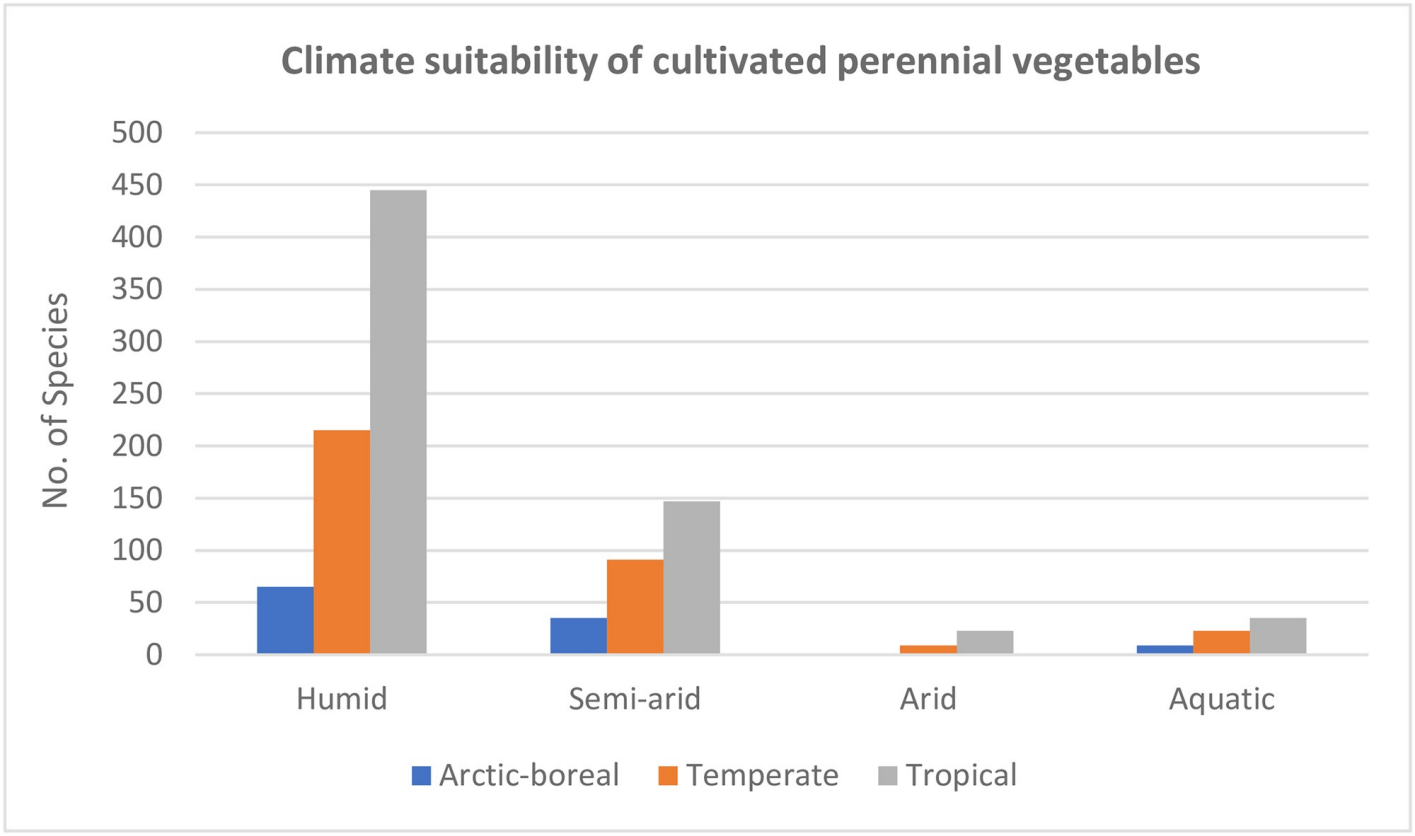
Climate suitability of cultivated perennial vegetables. “Arctic-boreal” includes arctic and boreal species (USDA hardiness zones 1–3), “temperate” includes warm and cold temperate species (USDA hardiness zones 4–8), “tropical” includes tropical lowlands (USDA zones 10–11, below 1500m elevation), tropical highlands (USDA zones 10–11, above 1500m elevation), and subtropics (USDA zone 9).

In order to assess the geographic variability of PV crops, we analyzed the full list of cultivated species to determine climate suitability. While PV species are suited to virtually every climate where crops are grown, diversity follows a familiar latitudinal gradient: increasing with humidity and temperature, from the poles toward the equator. Fig 3 shows the distribution of PV species for climate types.

Many PVs can be, in some cases must be, cultivated in shade. Nine percent of the 613 cultivated species identified in the study are suited to full shade, and 46% to partial shade.

### Carbon sequestration

The carbon sequestration impact of PV adoption under the 12 adoption scenarios as described in the methodology section is shown in Table 3. Total new adoption area of PVs ranges from 4.9–26.4 Mha. Carbon sequestration ranges from 22.7–280.6 MMT CO2-eq/yr in 2050.

**Table 3:** Global scenarios and sequestration impact of new PV adoption in 2050.

Scenario	Mha global vegetable production	Adoption Rate	% Woody PVs	Mha in Woody PVs	MMT CO2-eq/yr in Woody PVs	Mha in Non-Woody PVs	MMT CO2-eq/yr in Non-Woody PVs	Total Mha in PVs	Total MMT CO2-eq/yr PVs
1a	58.2	Linear	25%	1.2	4.6	3.7	5.8	4.9	22.7
1b	58.2	Linear	50%	2.5	33.8	2.5	3.9	4.9	37.7
1c	58.2	Linear	75%	3.7	50.7	1.2	1.9	4.9	52.6
2a	58.2	Exponential	25%	2.2	30.0	6.6	10.4	8.8	40.4
2b	58.2	Exponential	50%	4.4	60.0	4.4	6.9	8.8	67.0
2c	58.2	Exponential	75%	6.6	90.1	2.2	3.5	8.8	93.5
3a	174.5	Linear	25%	3.7	50.7	11.1	17.5	14.8	68.2
3b	174.5	Linear	50%	7.4	101.3	7.4	11.7	14.8	113.0
3c	174.5	Linear	75%	11.1	152.0	3.7	5.8	14.8	157.8
4a	174.5	Exponential	25%	6.6	90.1	19.8	31.2	26.4	121.2
4b	174.5	Exponential	50%	13.2	180.1	13.2	20.8	26.4	200.9
4c	174.5	Exponential	75%	19.8	270.2	6.6	10.4	26.4	280.6

This figure shows the carbon sequestration impact for each of 12 scenarios based on Mha new adoption of PVs by 2050, the percentage of woody PVs, and sequestration rates.

### Nutrition

In the comparison of PVs widely-grown reference vegetables, our results indicate that many PVs are high in the nutrients required to address widespread nutritional deficiencies, and certain species and subclasses of PVs are especially likely to be high in the key nutrients needed. The full set of data including those used in meta-analysis is included as S1 Data “Perennial Vegetable Nutrient Data” as an Excel spreadsheet.

**Fig 4:**
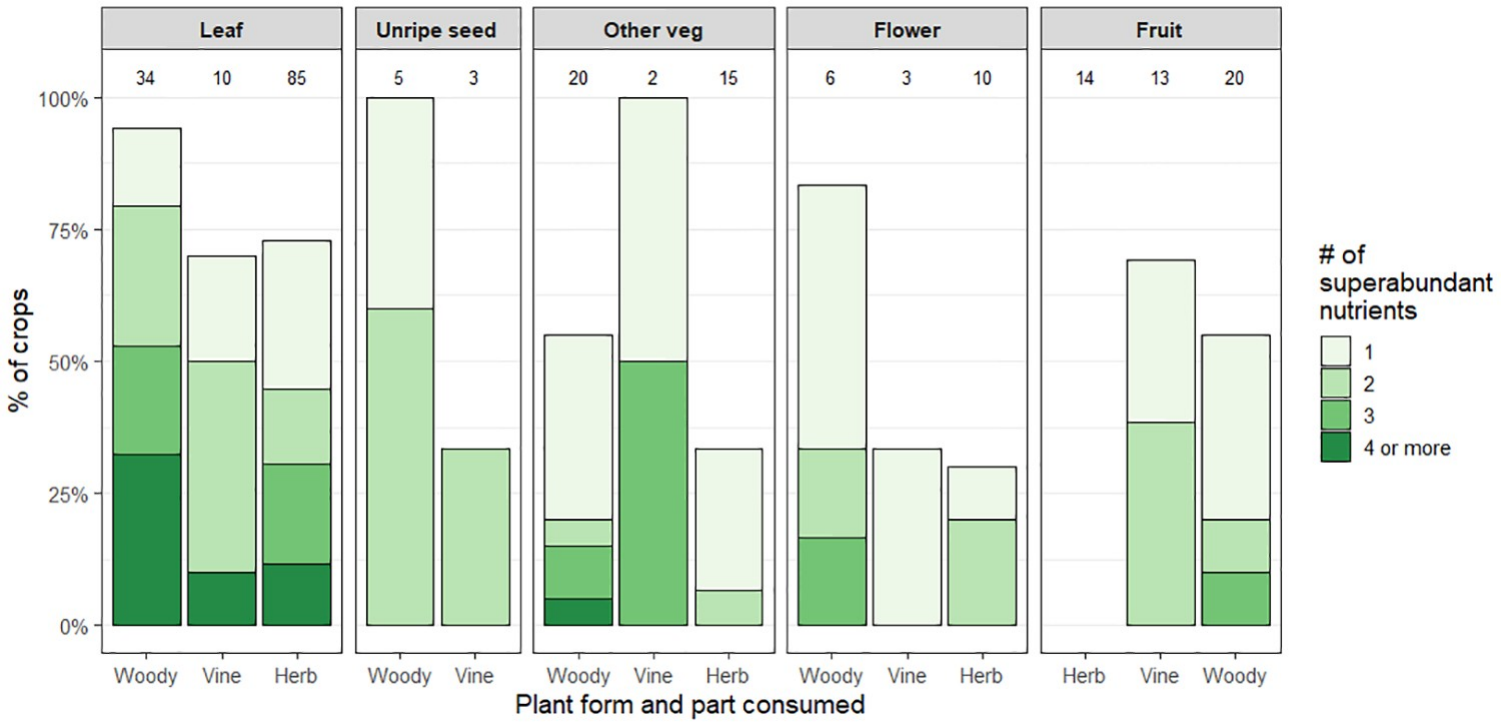
Proportions of perennial vegetables with one or more superabundant nutrients by plant form and part consumed. Nutrient levels are classified as superabundant when they are higher than the highest level of that nutrient among the reference vegetables. Here the percentage of crops with one or more superabundant nutrients is displayed for 240 perennial vegetables broken down by plant form and part used. The numbers of crops within each category are displayed along the top of the plot.

Of 240 PV species, 154 (64%) showed superabundant levels of nutrients (rated “very high” or “extremely high” as defined in Table 2). A full 23 PVs (10%) showed superabundant levels of four or more nutrients. The proportion of crops with superabundant nutrients across plant form and part used in displayed in Fig 4. Proportional levels of superabundant nutrients, especially of multiple nutrients, are most prominent in woody plants with edible leaves.

Table 4 shows the species with high levels of nutrients (annual and perennial) with the greatest potential to address traditional malnutrition. These nutrient levels are the results of meta-analysis, often from many sources, so references cannot be shown here. However, the “meta-analysis” sheet of the Perennial Vegetable Nutrition Data in the supplemental materials shows sources for all nutrition data. Note that several species only make the chart due to the combination of different plant parts. Woody perennials were the largest group in this category, with 62% of species being woody. They are followed by herbaceous perennials (25%), and vines (13%). Leaves are the part eaten for 81%, with leaves and flowers and/or fruit for 13% and fruit for 13%.

**Table 4:** Multi-nutrient species to address traditional malnutrition.

Name	Form	Thermal Climate	Moisture	Part	Fe	Zn	A	Folate
*Cnidoscolus aconitifolius*	Woody	Tropical	Humid, semi-arid, arid	Leaf	XH		XH	
*Malva sylvestris*	Perennial herb	Temperate, boreal/arctic	Humid	Leaf	XH	XH		
*Manihot esculenta*	Woody	Tropical	Humid, semi-arid	Leaf	XH	XH	VH	
*Momordica cochinchinensis*	Perennial vine	Tropical	Humid	Leaf, unripe fruit, fruit	VH	VH	VH	H
*Monochoria vaginalis*	Perennial herb	Tropical	Aquatic	Leaf	VH	VH	VH	
*Moringa oleifera*	Woody	Tropical	Humid, semi-arid	Leaf, unripe fruit, flowerbud	XH	VH	VH	
*Morus alba*	Woody	Tropical, temperate	Humid, semi-arid	Leaf	XH	XH	VH	
*Persicaria barbata*	Perennial herb	Tropical	Humid	Leaf	XH	VH		VH
*Pterocarpus mildbraedii*	Woody	Tropical	Humid	Leaf	XH	XH		
*Salix reticulata*	Woody	Boreal/arctic	Humid	Leaf	XH	XH		
*Senna obtusifolia*	Woody	Tropical	Humid	Leaf*	XH		XH	
*Senna sophera*	Woody	Tropical	Humid	Leaf	VH	VH	VH	H
*Solanum aethiopicum*	Perennial herb	Tropical*	Humid, semi-arid	Leaf	XH	VH	VH	
*Toona sinensis*	Woody	Tropical, temperate	Humid, semi-arid	Leaf	XH	XH	XH	
*Ulmus pumila*	Woody	Temperate, boreal/arctic	Humid, semi-arid, arid	Fruit	XH	XH		
*Vitis vinifera*	Perennial vine	Tropical, temperate, boreal/arctic	Humid, semi-arid	Leaf	VH	VH	VH	

“Tropical” indicates lowland tropics, highland tropics, and/or subtropics. “Temperate” indicates warm temperate and/or cold temperate. “Boreal/arctic” indicates boreal and/or arctic. “XH” indicates extremely high levels, over twice the highest level found in the reference crops. “VH” indicates very high levels between the highest level of reference crops and double the level of reference crops. “H” indicates high levels, in the highest tertile of the reference crops.

* Cultivated for its young leaves as a vegetable in four African countries, but mature leaves are a powerful laxative [36].

Table 5 shows the multi-nutrient species with the greatest potential to address industrial diet deficiencies. Note that several species only make the chart due to the combination of different plant parts and may or may not be ranked for an individual part. Note that several species only make the chart due to the combination of different plant parts. Woody perennials were again the largest group in this category, with 58% of species being woody. They are followed by herbaceous perennials (29%), and vines (13%). A majority (75%) feature leaves as the sole edible part, with 17% leaves with additional parts, and 8% shoots.

**Table 5:** Multi-nutrient species to address industrial diet deficiencies.

Name	Form	Thermal Climate	Moisture	Part	Fiber	Ca	Mg	A	C	E
*Asclepias syriaca*	Perennial herb	Temperate	Humid, semi-arid	Leaf		VH		VH	XH	
*Atriplex halimus*	Woody	Tropical, temperate	Humid semi-arid, arid	Leaf	VH	XH	XH			
*Bambusa polymorpha*	Woody	Tropical	Humid	Shoot	VH	VH	VH			
*Cnidoscolus aconitifolius*	Woody	Tropical	Humid semi-arid, arid	Leaf		VH	VH	XH	VH	
*Coccinia grandis*	Perennial vine	Tropical	Humid	Leaf, unripe fruit	VH			H		XH
*Dicliptera chinensis*	Perennial herb	Tropical	Humid	Leaf		VH		VH		XH
*Epilobium angustifolium*	Perennial herb	Temperate, boreal/arctic	Humid, semi-arid	Shoot	H	H	VH		VH	
*Gnetum gnemon*	Woody	Tropical	Humid	Leaf	VH		H	H	VH	H
*Limnocharis flava*	Perennial herb	Tropical	Aquatic	Leaf, stem, flowerbud	VH	XH	XH	H		
*Manihot esculenta*	Woody	Tropical	Humid, semi-arid	Leaf	H	VH		VH	XH	XH
*Momordica cochinchinensis*	Perennial vine	Tropical	Humid	Leaf, unripe fruit, fruit	H	VH		VH	XH	XH
*Moringa oleifera*	Woody	Tropical	Humid, semi-arid	Leaf, unripe fruit, flowerbud	H	VH	VH	VH	VH	H
*Morus alba*	Woody	Tropical, temperate	Humid, semi-arid	Leaf	VH	VH	VH	VH	VH	
*Pisonia umbellifera*	Woody	Tropical	Humid	Leaf	VH	VH	VH			
*Sauropus androgynus*	Woody	Tropical	Humid	Leaf				VH	VH	XH
*Senna obtusifolia*	Woody	Tropical	Humid	Leaf*		VH		XH	VH	
*Senna sophera*	Woody	Tropical	Humid	Leaf		H		VH	VH	VH
*Sesbania grandiflora*	Woody	Tropical	Humid	Leaf	XH	VH	VH		H	H
*Silene vulgaris*	Perennial herb	Temperate, boreal/arctic	Humid, semi-arid	Leaf	VH			VH		XH
*Solanum aethiopicum*	Perennial herb	Tropical*	Humid, semi-arid	Leaf		VH		VH		XH
*Toona sinensis*	Woody	Tropical, temperate	Humid, semi-arid	Leaf		VH		XH	VH	XH
*Trichanthera gigantea*	Woody	Tropical	Humid	Leaf		XH	XH			
*Urtica dioica*	Perennial herb	Temperate, boreal/arctic	Humid	Leaf	H	VH	H		VH	XH
*Vitis vinifera*	Perennial vine	Tropical, temperate	Humid, semi-arid	Leaf	XH	VH	VH	VH		H

“Tropical” indicates lowland tropics, highland tropics, and/or subtropics. “Temperate” indicates warm temperate and/or cold temperate. “Boreal/arctic” indicates boreal and/or arctic. *Perennial frequently grown as an annual. “XH” indicates extremely high levels, over twice the highest level found in the reference crops. “VH” indicates very high levels between the highest level of reference crops and double the level of reference crops. “H” indicates high levels, in the highest tertile of the reference crops.

* Cultivated for its young leaves as a vegetable in four African countries, but mature leaves are a powerful laxative [36].

For each nutrient, the ten crops with highest concentrations were ranked. See S2 Table “Top Ten Species by Nutrient Concentration” for results. Of these species with at least one elite-level nutrient concentration, 52% are woody, 33% are herbaceous perennials, and 15% are vines. For the part used, 64% are leaves, 13% are fruits and unripe seeds, 7% are flowers or flowerbuds, and 5% are shoots.

## Discussion

PVs are highly a highly diverse and underutilized class of crop plants. They offer impressive potential to address nutrient deficiencies impacting billions of people around the world. PVs sequester carbon, and increased adoption to provide nutrition would offer this important cobenefit. Their suitability to a wide range of climates, as well as for growing conditions adverse to annual vegetables, indicates a potential for scaling up production across a broad geographic range.

Carbon sequestration impacts are variable depending on the assumptions used. The mitigation potential of PVs will be greater if a) global vegetable area is increased to be sufficient to meet nutrient needs and b) an emphasis is placed on woody PVs rather than vines and herbaceous perennials.

PVs offer a modest but important contribution to agricultural climate change mitigation on vegetable cropland. Potential global impact of improved management of all global cropland is estimated at 1,400–2,300 MMT CO2-eq by 2050 [14]. As 3.5% of world cropland is in vegetable production currently [6], the mitigation potential for this land is 49–80 MMT CO2-eq/yr in 2050 under improved management. Increased PV adoption would sequester 22.7–280.6 MMT CO2-eq in 2050, demonstrating a notable improvement in carbon dioxide removal and storage in soils and perennial biomass via the partial perennialization of vegetable production.

Perennial crops, as a class, are not more nutritious than annual crops as a class. Indeed some annual crops (notably the *Amaranthus* species) greatly exceed the nutrition of the reference crops, clearly standing out for their ability to address nutrient deficiencies. However, the nutrition of perennials as a class, and of many individual perennial crops, certainly outperforms the reference vegetables tracked by FAO (the reference vegetables include both annuals and perennials, but only those that are widely grown and traded). The high frequency of superabundance in woody PVs is clearly shown in Fig 4.

Regarding the top ten species for each key nutrient, the superior performance of perennial species is notable, particularly woody perennials. It is also noteworthy that, of the crops tracked by FAO, only cassava leaf makes it onto these lists. It would appear that the vegetables which are most widely grown and marketed are not those best suited to addressing nutrient deficiencies. An exception is folate, for which few vegetables, annual or perennial, exceeded the set of reference vegetables. Folate, however, is also the least-reported nutrient of the nine nutrients we examined (81 data points, compared with 221 for calcium).

Some species are found on the multi-nutrient tables for both traditional malnutrition and industrial diet deficiencies. These species represent extremely powerful tools to address nutrient deficiencies. They include the woody plants *Cnidoscolus aconitifolius*, *Manihot esculenta*, *Moringa oleifera*, *Morus alba*, *Senna obtusifolia*, *S*. *sophera*, and *Toona sinensis*, the perennial vines *Momordica cochinchinensis* and *Vitis vinifera*, and the perennial herb *Solanum aethiopicum*.

The meta-analysis approach taken in this study does not account for differences between variety, climate, farming system and practice, and soils, all of which can impact nutritional composition. [67]. Further research should account for and determine the reasons for variation within species.

Data on sequestration rates of PV species, in various production systems, are needed. This would allow improved climate impact projections.

## Conclusions

Perennial vegetables represent a very large and neglected group of crops. They have potential to increase carbon sequestration in vegetable production and address nutrient deficiencies affecting over 2 billion people. They offer an important set of tools to tackle some of the key challenges of the 21st century.

This study found that the vegetables which are most widely grown and marketed are not those best able to address nutrient deficiencies, with a few exceptions. As world vegetable production needs to be tripled to provide healthy diets for all, efforts should be made to incorporate PVs in the new production areas.

It is especially good news that tree vegetables are among the most nutritious, feature high sequestration rates, and represent over a quarter of PV species. Note that tree vegetables are not confined to the tropics, with multiple species for colder climates, even including cold drylands.

The identification of species with high levels of multiple key nutrients offers to potential to highlight these crops in development and nutrition campaigns. Regional programs could identify species suited to the climate and targeted to the specific nutrient needs of the population. Given the wide distribution of PVs, species native to a given region can be prioritized.

Perennial vegetables can play a role in the perennialization of agriculture to increase carbon sequestration in soil and perennial biomass. Their potential for use in agroforestry systems, especially given the abundance of shade-tolerant PV species, is notable given the high sequestration rates of agroforestry systems. It should be noted that some PVs sequester far more carbon than others, notably the full-sized woody plants like trees, palms, and bamboos.

Time and time, again around the world, over the course of thousands of years, farmers and gardeners have taken these species into cultivation. Their highly desirable biodiversity, carbon sequestration, and nutrition impacts should be a major motivation to research, cultivate, and promote PV crops. This study is intended as a launching point for initiatives to increase the adoption and utilization of these remarkable crops.

## Supporting information

S1 Table Cultivated perennial vegetable species of the world. An inventory of 613 cultivated perennial vegetable species with form, part used, and climate suitability.
(XLSX)

S2 Table Perennial vegetable nutrition. Mata-analysis of data on nutrition of 240 annual and perennial vegetable species.
(DOCX)

S1 Data Top ten species by nutrient concentration. Tables of the ten vegetable species highest in each of the key nutrients for addressing traditional malnutrition and industrial diet deficiencies.
(XLSX)

S1 Fig Nutritional data acquired by plant form and part used. This figure displays the number of data points acquired for each nutrient, their distribution across crops by plant form and part used. Numbers in parenthesis on the vertical axis represent the number of crops in that category. Numbers in parenthesis on the horizontal axis represent the total number of data points for that nutrient. Numbers in cells show the number of data points of a given nutrient for a given category of crops. Fill color in each cell represents the proportion of crops in the given category for which data on the given nutrient is present.
(TIF)
