# Supplementary material for: Perennial vegetables: A neglected resource for biodiversity, carbon sequestration, and nutrition
Source: PLoS One. 2020 Jul 10;15(7):e0234611. doi: 10.1371/journal.pone.0234611 (PMC7351156; doi:10.1371/journal.pone.0234611)
Supplement: S2 Table — Mata-analysis of data on nutrition of 240 annual and perennial vegetable species. (DOCX) [file pone.0234611.s002.docx]

**Perennial vegetables: A neglected resource for biodiversity, carbon sequestration, and nutrition**

Eric Toensmeier, Rafter Ferguson, Mamta Mehra

**Supplemental materials: Top ten species by nutrient concentration**

**Top ten species by nutrient: Fiber**

| **Latin name** | **Form** | **Part Used** | **% Fiber** |
| --- | --- | --- | --- |
| *Phaseolus coccineus* | Perennial vine | Unripe seed | 12.2% |
| *Vitis vinifera* | Perennial vine | Leaf | 11.0% |
| *Parmentiera aculeata* | Woody | Fruit | 10.6% |
| *Solanum torvum* | Woody | Fruit | 9.06% |
| *Dacryodes edulis* | Woody | Fruit | 8.2% |
| *Sesbania grandiflora* | Woody | Leaf | 7.8% |
| *Balanites aegyptiaca* | Woody | Leaf | 6.8% |
| *Gundelia tournefortii* | Herbaceous perennial | Flowerstalk | 6.4% |
| *Gnetum gnemon* | Woody | Leaf | 6.3% |
| *Dystaenia takesimiana* | Herbaceous perennial | Leaf | 5.8% |

**Top ten species by nutrient: Calcium**

| **Latin name** | **Form** | **Part Used** | **Ca mg/100g** |
| --- | --- | --- | --- |
| *Trichanthera gigantea* | Woody | Leaf | 1,107.5 |
| *Atriplex halimus* | Woody | Leaf | 912.0 |
| *Limnocharis flava* | Herbaceous perennial | Flowerbud | 770.9 |
| *Morus alba* | Woody | Leaf | 648.0 |
| *Erythrina variegata* | Woody | Leaf | 639.1 |
| *Piper sarmentosum* | Herbaceous perennial | Leaf | 601.0 |
| *Sesbania grandiflora* | Woody | Leaf | 577.5 |
| *Psophocarpus scandens* | Perennial vine | Leaf | 565.0 |
| *Bambusa polymorpha* | Woody | Shoot | 560.0 |
| *Solanum aethiopicum* | Herbaceous perennial | Leaf | 523.0 |

**Top ten species by nutrient: Iron**

| **Latin name** | **Form** | **Part Used** | **Fe mg/100g** |
| --- | --- | --- | --- |
| *Viola odorata* | Herbaceous perennial | Flower | 39.0 |
| *Lemna spp.* | Herbaceous perennial | Leaf | 30.3 |
| *Atriplex halimus* | Woody | Leaf | 18.6 |
| *Morus alba* | Woody | Leaf | 14.4 |
| *Saccharum spontaneum* var. *edulis* | Herbaceous perennial | Flowerbud | 9.3 |
| *Pentarrhinum insipidum* | Perennial vine | Leaf | 8.7 |
| *Canavalia gladiata* | Perennial vine | Unripe fruit | 8.6 |
| *Limnophila rugosa* | Herbaceous perennial | Leaf | 8.2 |
| *Ulmus pumila* | Woody | Fruit | 7.9 |
| *Piper sarmentosum* | Herbaceous perennial | Leaf | 7.6 |

**Top ten species by nutrient: Magnesium**

| **Latin name** | **Form** | **Part Used** | **Mg mg/100g** |
| --- | --- | --- | --- |
| *Atriplex halimus* | Woody | Leaf | 610.0 |
| *Limnocharis flava* | Herbaceous perennial | Flowerbud | 228.1 |
| *Trichanthera gigantea* | Woody | Leaf | 174.7 |
| *Marsdenia australis* | Perennial vine | Fruit | 172.2 |
| *Salix reticulata* | Woody | Leaf | 167.0 |
| *Pisonia umbellifera* | Woody | Leaf | 165.0 |
| *Persicaria barbata* | Herbaceous perennial | Leaf | 160.0 |
| *Morus alba* | Woody | Leaf | 159.0 |
| *Bambusa polymorpha* | Woody | Shoot | 140.0 |
| *Moringa oleifera* | Woody | Leaf | 112.0 |

**Top ten species by nutrient: Zinc**

| **Latin name** | **Form** | **Part Used** | **Zn mg/100g** |
| --- | --- | --- | --- |
| *Chrysanthemum indicum* | Herbaceous perennial | Leaf | 7.8 |
| *Pterocarpus mildbraedii* | Woody | Leaf | 5.0 |
| *Hosta sieboldii* | Herbaceous perennial | Leaf | 4.2 |
| *Salix arctica* | Woody | Leaf | 4.1 |
| *Hosta sieboldiana* | Herbaceous perennial | Leaf | 3.7 |
| Unspecified palm shoot | Woody | Shoot | 3.7 |
| *Hosta longipes* | Herbaceous perennial | Leaf | 3.6 |
| *Salix reticulata* | Woody | Leaf | 3.5 |
| *Gundelia tournefortii* | Herbaceous perennial | Flowerstalk | 3.5 |
| *Ulmus pumila* | Woody | Fruit | 3.3 |

**Top ten species by nutrient: Vitamin A**

| **Latin name** | **Form** | **Part Used** | **RAE mg/100g** |
| --- | --- | --- | --- |
| *Lycium barbatum* | Woody | Fruit | 4.3 |
| *Toona sinensis* | Woody | Leaf | 1.9 |
| *Cnidoscolus aconitifolius* | Woody | Leaf | 1.4 |
| *Senna obtusifolia* | Woody | Leaf | 1.3 |
| *Momordica cochinchinensis* | Perennial vine | Leaf | 1.0 |
| *Manihot esculenta* | Woody | Leaf | 1.0 |
| *Senna sophora* | Woody | Leaf | 1.0 |
| *Salix pulchra* | Woody | Leaf | 0.9 |
| *Sauropus androgynus* | Woody | Leaf | 0.9 |
| *Asystasia gangetica* | Herbaceous perennial | Leaf | 0.8 |

**Top ten species by nutrient: Folate**

| **Latin name** | **Form** | **Part Used** | **B9 mcg/100g** |
| --- | --- | --- | --- |
| *Solanum torvum* | Woody | Fruit | 327.0 |
| *Beta vulgaris maritima* | Herbaceous perennial | Leaf | 302.0 |
| *Persicaria barbata* | Herbaceous perennial | Leaf | 287.0 |
| *Silene vulgaris* | Herbaceous perennial | Leaf | 267.0 |
| *Osmunda japonica* | Herbaceous perennial | Shoot | 210.0 |
| *Allium hookeri* | Herbaceous perennial | Leaf | 177.0 |
| *Cajanus cajan* | Woody | Unripe seed | 173.0 |
| *Kalimeris indica* | Herbaceous perennial | Leaf | 170.0 |
| *Aralia elata* | Woody | Leaf | 160.0 |
| *Humulus lupulus* | Perennial Vine | Shoot | 144.0 |

**Top ten species by nutrient: Vitamin C**

| **Latin name** | **Form** | **Part Used** | **Ascorbic Acid mg/100g** |
| --- | --- | --- | --- |
| *Momordica cochinchinensis* | Perennial vine | Leaf | 935.3 |
| *Yucca gigantea* | Woody | Flower | 393.0 |
| *Asclepias syriaca* | Herbaceous perennial | Leaf | 391.0 |
| *Manihot esculenta* | Woody | Leaf | 273.8 |
| *Momordica cochinchinensis* | Perennial vine | Ripe fruit | 258.9 |
| *Momordica dioica* | Perennial vine | Unripe fruit | 248.6 |
| *Morus alba* | Woody | Leaf | 220.0 |
| *Moringa oleifera* | Woody | Leaf | 218.6 |
| *Cnidoscolus aconitifolia* | Woody | Leaf | 216.7 |
| *Urtica dioica* | Herbaceous perennial | Leaf | 208.5 |

**Top ten species by nutrient: Vitamin E**

| **Latin name** | **Form** | **Part Used** | **Vitamin E mg/100g** |
| --- | --- | --- | --- |
| *Toona sinensis* | Woody | Leaf | 19.9 |
| *Humulus lupulus* | Perennial vine | Shoot | 14.4 |
| *Urtica dioica* | Herbaceous perennial | Leaf | 14.4 |
| *Manihot esculenta* | Woody | Leaf | 13.1 |
| *Silene vulgaris* | Herbaceous perennial | Leaf | 11.3 |
| *Lycium chinense* | Woody | Leaf | 8.9 |
| *Angelica keiskei* | Herbaceous perennial | Leaf | 7.6 |
| *Solanum aethiopicum* | Herbaceous perennial | Leaf | 7.3 |
| *Sauropus androgynus* | Woody | Leaf | 6.2 |
| *Anredera cordifolia* | Perennial vine | Leaf | 6.1 |
