# Supplementary figures and images for: Perennial vegetables: A neglected resource for biodiversity, carbon sequestration, and nutrition
Source: PLoS One. 2020 Jul 10;15(7):e0234611. doi: 10.1371/journal.pone.0234611 (PMC7351156; doi:10.1371/journal.pone.0234611)

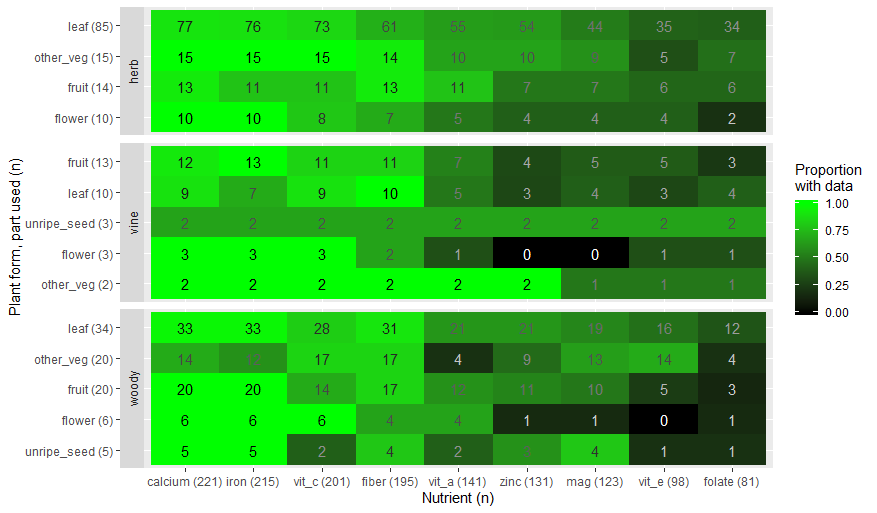

Supplement: S1 Fig — This figure displays the number of data points acquired for each nutrient, their distribution across crops by plant form and part used. Numbers in parenthesis on the vertical axis represent the number of crops in that category. Numbers in parenthesis on the horizontal axis represent the total number of data points for that nutrient. Numbers in cells show the number of data points of a given nutrient for a given category of crops. Fill color in each cell represents the proportion of crops in the given category for which data on the given nutrient is present. (TIF) [file pone.0234611.s004.tif]
